# IFN-γ surmounts PD-L1/PD1 inhibition to CAR-T cell therapy by upregulating ICAM-1 on tumor cells

**DOI:** 10.1038/s41392-020-00357-7

**Published:** 2021-01-07

**Authors:** E Dong, Xiao-zhu Yue, Lin Shui, Bin-rui Liu, Qi-qi Li, Yun Yang, Hui Luo, Wei Wang, Han-shuo Yang

**Affiliations:** State Key Laboratory of Biotherapy and Cancer Center, West China Hospital, Sichuan University and Collaborative Innovation Center for Biotherapy, 610041 Chengdu, China

**Keywords:** Adaptive immunity, Immunotherapy

**Dear Editor,**

Chimeric antigen receptor modified T (CAR-T) cell therapy has shown potent antitumor activity against relapsed and refractory hematological malignancies. However, its efficacy in solid tumors is limited,^1^ partly because of the inhibition of PD-L1/PD-1 signaling on CAR-T cells in solid tumors. Further optimizations of CAR-T cells by disrupting PD-1 signaling have improved the anti-tumor efficacy of CAR-T cells.^2^ Here, we report an alternative approach that sensitizes tumor cells by interferon (IFN)-γ, but without modifying T cells; this strategy surmounts PD-1 inhibition and markedly enhances CAR-T anti-tumor activities in vitro and in vivo.

We constructed two CAR-T cells containing CD28 or 4-1BB co-stimulatory receptor intracellular domains in tandem with CD3$\zeta$ that target the tumor antigens of HER2 and mesothelin (MSLN) (Supplementary Fig. S1a). Both HER2 and MSLN CAR-T cells exhibited high rates of cytotoxicity to targeted tumor cells, with robust secretion of IFN-γ and interleukin (IL)-2 (Supplementary Fig. S1b, c) and dramatically increased expression of effector activity surface molecule CD107a and intracellular granzyme B (Supplementary Fig. S1d). PD-L1 was significantly upregulated on tumor cells, and coinhibitory molecules including PD-1, TIM-3, and LAG-3 were markedly elevated on CAR-T cells (Supplementary Fig. S2a–c). Moreover, the recursive antigen encountering of HER2 CAR-T cells, mimicking the in vivo antitumor milieu where CAR-T cells serially encounter and kill tumor cells (Supplementary Fig. S3a), showed high cytotoxicity and IFN-γ release (Supplementary Fig. S3b), despite high PD-L1 expression on almost all tumor cells (Supplementary Fig. S3c). These results indicated the existence of an intrinsic mechanism of CAR-T cells that surmounts the inhibitory effects of PDL1/PD-1 during tumor cell killing.

IFN-γ is an important inducer of PD-L1 expression on tumor cells.^3^ To evaluate the effects of IFN-γ-induced PD-L1 expression on CAR-T cytotoxicity, tumor cells were primed with IFN-γ for 24 h before exposure to CAR-T cells. IFN-γ concentration was optimized as <10 ng/ml to ensure induction of PD-L1 expression without inhibiting tumor cell growth (Supplementary Fig. S4a, b). Notably, however, CAR-T cells showed more potent cytolytic activity and IFN-γ secretion when exposed to IFN-γ-primed tumor cells with high expression of PD-L1 (Fig. 1a, b). Conversely, when IFN-γ was neutralized by anti-IFN-γ antibody, the cytotoxicity of CAR-T cells was significantly attenuated (Fig. 1c), which indicates that IFN-γ was critical to CAR-T activities. Notably, in these tests, the medium was replaced with fresh medium to eliminate residual IFN-γ (<10 pg/ml, data not shown), excluding the direct effect of IFN-γ on CAR-T cells. We thus speculated that IFN-γ functioned to CAR-T activity by acting on tumor cells. To examine this possibility, we first primed tumor cells with low concentrations of IFN-γ and measured the cytotoxicity of CAR-T in fresh medium containing sufficient anti-IFN-γ antibody to neutralize IFN-γ from CAR-T (Fig. 1d). CAR-T cells still exhibited similar killing activity to IFN-γ-primed tumor cells when IFN-γ was neutralized (Fig. 1e). Second, we disrupted the IFN-γR2 gene (IFNGR2) in tumor cells using the CRISPR-Cas9 system to block IFN-γ signaling (Supplementary Fig. S5a–c), because IFN-γR2 has been shown to determine the extent of IFN-γ-induced signaling in given cell populations.^4^ IFN-γR2-null tumor cells grew similarly to control cells (Supplementary Fig. S5d) and did not express PD-L1 any more under IFN-γ treatment (Supplementary Fig. S5e). However, these cells were more resistant to the cytolysis of CAR-T cells (Fig. 1f). The enhancement of CAR-T cytotoxicity by IFN-γ priming was also significantly diminished as shown by two different target tumor cell lines (Fig. 1g). These results indicated that IFN-γ on tumor cells is involved in the intrinsic mechanism of CAR-T to circumvent PD-L1/PD-1.

**Fig. 1:**
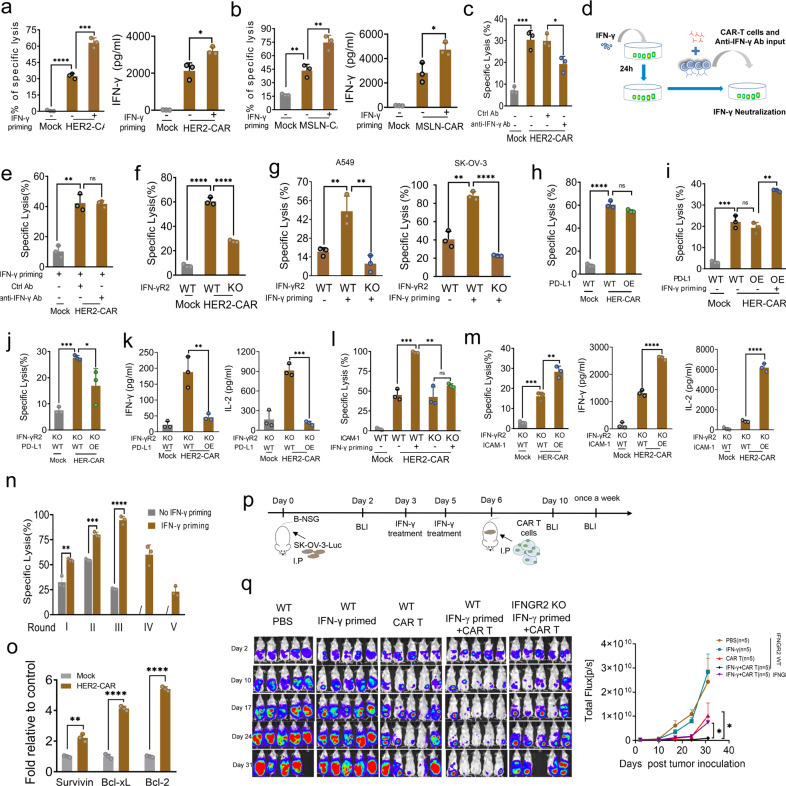
IFN-γ enhances CAR-T anti-tumor activities by sensitizing tumor cells expressing ICAM-1. **a**, **b** IFN-γ priming to tumor cells enhances the specific cytolytic ability and IFN-γ secretion of HER2-CAR (**a**) and MSLN-CAR T (**b**). **c** The blockage to IFN-γ by using neutralizing antibody decreases the lytic ability of HER2-CAR T cells. **d**, **e** Tumor cells were primed by IFN-γ for 24 h, then were washed using fresh culture media to remove IFN-γ. Sufficient anti-IFN-γ neutralizing antibody was used to block IFN-γ release by CAR-T cells (**d**). The specific lysis was measured by LDH assay (**e**). **f** IFN-γR2 knockout in tumor cells significantly decreased CAR-T cytotoxicity. **g** IFN-γR2 knockout impaired the enhancement of IFN-γ-priming to CAR-T cytotoxicities. **h** PD-L1-overexpression did not decrease CAR-T cytotoxicity to tumor cells. **i** IFN-γ-priming enhanced CAR-T-specific lysis to tumor cells overexpressing PD-L1. **j**, **k** PD-L1-overexpression in IFN-γR2-deficient tumor cells inhibited CAR-T cytotoxicity (**j**) and cytokines release (**k**). **l** The knockout of ICAM-1 in tumor cells impaired the enhancement of IFN-γ-priming to CAR-T cytotoxicity. **m** The overexpression of ICAM-1 in IFN-γR2 KO tumor cells enhanced CAR-T cytotoxicity and the secretion of IFN-γ and IL-2. **n** The enhancement of IFN-γ priming to the serial cytotoxic ability of CAR-T cells. **o** The relatively increased RNA expression of Bcl-2, Bcl-xl, survivin in CAR-T cells that encountered IFN-γ-primed tumor cells. **p** The procedure of combinational therapy of IFN-γ and HER2-CAR T cells in peritoneal ovarian cancer models in B-NSG mice. IFN-γ was administrated (25 μg per mouse) prior to CAR-T cells. IP, intraperitoneal injection. **q** Bioluminescent images and quantitative analysis of peritoneal metastases of human ovarian cancer in selected timepoints during the experiments. Bars represent the mean ± s.e.m. *p-*values determined by unpaired two-tailed *t*-tests. *p < 0.05, **p < 0.01, ***p < 0.001

To directly characterize the function of IFN-γ in surmounting PD-L1/PD-1, we tested CAR-T activities on tumor cells that stably overexpress PD-L1. Overexpression of PD-L1 on tumor cells (Supplementary Fig. S6) did not render tumor cells resistant to CAR-T killing (Fig. 1h), and also did not impair the enhancement of CAR-T cytotoxicity to IFN-γ-primed tumor cells (Fig. 1i). Notably, however, PD-L1 overexpression in IFN-γR2-null tumor cells significantly suppressed both cytolytic activity (Fig. 1j) and secretion of IFN-γ and IL-2 (Fig. 1k). These results indicated that IFN-γ signaling on tumor cells enhances CAR-T activities by surmounting the inhibitory effects of PD-L1/PD-1. In contrast, the inhibitory function of PD-L1/PD-1 on CAR-T activity requires the absence of IFN-γ signaling in tumor cells. This is consistent with the previous study that defects of the interferon signaling pathway are associated with acquired resistance to immune checkpoint blockade therapy.^5^

We next investigated how IFN-γ surmounts PD-L1/PD-1 inhibitory effects on CAR-T. IFN-γ induces complex molecular events beyond PD-L1 overexpression in tumor cells. We first examined whether IFN-γ increased HER2 expression. However, HER2 expression was decreased in IFN-γ-primed tumor cells (Supplementary Fig. S7), which indicates that HER2 was not involved in IFN-γ-mediated effects on CAR-T. We found that IFN-γ induced intercellular adhesion molecule 1 (ICAM-1, also known as CD54) overexpression on multiple kinds of solid tumor cells (Supplementary Fig. S8a), and LFA-1 (the β2 integrin adhesion molecule) was significantly elevated on CAR-T cells after tumor cell exposure (Supplementary Fig. S8b). ICAM-1 is a cell surface glycoprotein on antigen-presenting cells (APCs) and plays a critical role during an effective immune response. The interaction of antigen-laden APCs with CD8 T cells is mediated by the interaction of ICAM-1 to its receptor LFA-1 on T cells. The ICAM-1/LFA-1 interaction promotes efficient adhesion of T cells with APCs and transmits intracellular signals to promote T-cell activation and proliferation.^6^ We then investigated the function of ICAM-1 on CAR-T activity. Knockout of ICAM-1 (Supplementary Fig. S9) in tumor cells with functional IFN-γ receptor almost completely abolished the increase of CAR-T cytotoxicity to IFN-γ-primed tumor cells (Fig. 1l), which indicates the critical role of ICAM-1 for CAR-T activity. We also ectopically overexpressed ICAM-1 on tumor cells (Supplementary Fig. S10); however, it did not promote the specific cytolysis of CAR-T to tumor cells (Supplementary Fig. S11). We speculated this was because of the elevation of ICAM-1 induced by IFN-γ from activated CAR-T cells. We then overexpressed ICAM-1 in IFN-γR2-knockout cells and found that ICAM-1 overexpression made IFN-γR2-deficient tumor cells more vulnerable to CAR-T killing and more cytokine release (Fig. 1m). Altogether, these results indicated that IFN-γ-induced ICAM-1 expression underlies the mechanism by which IFN-γ surmounts PD-L1/PD-1 to sustain or enhance CAR-T anti-tumor activities.

We next tested whether IFN-γ priming could help CAR-T achieve better therapeutic effects to solid tumors. In vitro serial killing experiments showed that CAR-T cells maintained cytolytic activity to IFN-γ-primed tumor cells until round five, compared with round three for control tumor cells (Fig. 1n). Anti-apoptosis genes including bcl-2, bcl-xl, and survivin were significantly upregulated in CAR-T cells that encountered IFN-γ-primed tumor cells (Fig. 1o). Next, we established a peritoneal ovarian cancer model in NSG mice and administrated IFN-γ twice before CAR-T cells were intraperitoneally injected (Fig. 1p). Bioluminescence imaging showed that IFN-γ was ineffective in inhibiting tumor growth, while CAR-T only delayed tumor growth. However, IFN-γ pretreatment combined with CAR-T cells led to elimination of tumors in three of five mice. The remaining two tumors were much smaller than controls (Fig. 1q). However, when IFN-γR2 was deficient, tumors were resistant to the combination therapy. Collectively, these in vitro and in vivo results indicated that sequential therapy using IFN-γ and CAR-T would be an effective strategy for solid tumors in the clinic.

In summary, we demonstrated that although IFN-γ induces PD-L1 expression in tumor cells, IFN-γ is indispensable for CAR-T cell activities by inducing ICAM-1 expression to surmount PD-L1/PD-1. The sequential therapy of IFN-γ with CAR-T could achieve better therapeutic effects in solid tumors. While current efforts to improve CAR-T therapy to solid tumors have focused on modification of T cells, here we propose that sensitizing tumor cells is also an effective and feasible approach.

## Supplementary information

IFN-γ enhances CAR-T anti-tumor activities by sensitizing tumor cells expressing ICAM-1

## Data Availability

The authors declare that the data supporting the findings of this study are available within the paper and its supplementary information files.
