# Supplementary material for: IFN-γ surmounts PD-L1/PD1 inhibition to CAR-T cell therapy by upregulating ICAM-1 on tumor cells
Source: Signal Transduct Target Ther. 2021 Jan 7;6:20. doi: 10.1038/s41392-020-00357-7 (PMC7811529; doi:10.1038/s41392-020-00357-7)
Supplement: Supplementary file 1 — IFN-γ enhances CAR-T anti-tumor activities by sensitizing tumor cells expressing ICAM-1 [file 41392_2020_357_MOESM1_ESM.pdf]

Supplementary Materials for

IFN-γ Surmounts PD-L1/PD1 inhibition to CAR-T by up-regulating ICAM-1 on tumor cells

E Dong[1], Xiao-zhu Yue[1], Lin Shui[1], Bin-rui Liu[1], Qi-qi Li[1], Yun Yang[1], Hui Luo[1], Wei Wang*, Han-shuo Yang[1]*

1. State Key Laboratory of Biotherapy and Cancer Center, West China Hospital, Sichuan University and Collaborative Innovation Center for Biotherapy, Chengdu 610041, China.

*Correspondence should be addressed Hanshuo Yang (yhansh@scu.edu.cn) and Wei Wang (weiwang@scu.edu.cn).

This PDF file includes:

Materials and Methods;

References for 'Materials and Methods';

Figure. s1-s11.

Material and methods

Cell lines

The human epithelial ovarian cancer cell line SK-OV-3, lung cancer cell line A549 and lentivirus packaging cell line HEK 293TD were obtained from American Type Culture Collection (ATCC;Manassas, VA, USA). All the cell lines were cultured in Dulbecco's modified Eagle's medium (thermo fisher scientific) supplemented with 10% heat-inactivated FBS and 1% penicillin and streptomycin. All cell lines were maintained in a humidified incubator with 95% air and 5% CO_2 at 37°C.

DNA constructs and lentivirus production

The extracellular fragment was composed of HER2-specific scFv from HER2, linked to human CD8α hinge region, transmembrane domain of the CD8α. Intracellular region included 4-1BB cytoplasmic domain and CD3ζ molecule, simply called HER2BBZ (HER2-CAR) was cloned into lentivirus-vector pWPXLd. Another CAR targeting to mesothelin with CD28 cytoplasmic and CD3 ζ molecule was termed as MSLN28Z(MSLN-CAR), all components were cloned into lentivirus-vector pWPXLd. The subsequent lentivirus production, concentration and quantification were completed in our lab. Tumor cells were engineered to express enhanced firefly luciferase (ffluc) by transduction with lentivirus carrying the ffluc gene under the control of the CMV promoter. To construct a recombinant eukaryotic vector bearing the gene of human ICAM-1(CD54), the vector pWPXLd was used to construct pWPXLd -ICAM-1 using the restriction sites BamHI and EcoRI. The vector pCDH expressing the human PD-L1 gene was cloning by EcoRI and XbaI restriction site. Knockout cell lines were generated using the CRISPR–Cas9 system. To generate knockout IFNGR2，ICAM-1 cells, cells were transduced with pLentiCRISPRV2 vector (Addgene) encoding the annealed double-stranded single-guide RNAs(sgRNAs) targeting IFNGR2 and ICAM-1 by BsmB I (NEB). The sgRNA sequence TCGCCTGTACAACGCAGAGC(sgRNA IFNGR2#1) and GGACCTGCTCTGCGT TGTAC(sgRNA IFNGR2#2) were used for generating IFNGR2 KO cells. The sgRNA sequences GCGGCTGACGTGTGCAGTAA(sgRNA ICAM-1#1) and CAACTTGTC AGCCCCCGGGT(sgRNA ICAM-1#2) were used to generate bulk populations of ICAM-1 KO cells. Lentivirus was generated as previously described.[1] Briefly, 293T cells were transfected with packaging plasmid psPAX2, PMD2.0G (Invitrogen)and CAR lentiviral backbone plasmid using a modified calcium phosphate method. Viral supernatants were collected after transfection 48 and 72 hours and concentrated through ultracentrifugation at 70,000g for 120 minutes. Lentiviral pellets were resuspended in phosphate-buffered saline (PBS)-lactose solution (4 g lactose per 100 mL PBS), aliquoted and stored at −80°C. Lentiviral titers were quantified using 293T cells based on CAR expression.

T cell isolation, lentiviral transduction, and ex vivo expansion

Human peripheral blood mononuclear cells (PBMCs) were isolated from consented research participants (healthy donors) under protocols approved by Ethics Committee of the State Key Laboratory of Biotherapy. On the day of leukapheresis, peripheral blood mononuclear cells (PBMC) were isolated by density gradient centrifugation over Ficoll-Paque (GE Healthcare) followed by multiple washes in DPBS (thermo fisher scientific). Freshly isolated PBMC were then cultured in complete X-VIVO 15 medium (Lonza) containing 100 U/mL recombinant human IL-2 (peprotech) and 5% human AB serum (Sigma-Aldrich) and stimulated with anti-CD3 and anti-CD28 magnetic beads for 24-36 hours (Life Technologies). For CAR lentiviral transduction, desired lentivirus at a multiplicity of infection of 5-10 were added to each retronectin-coated well plates and centrifuged at 1000g for 2 hours at 32℃. After centrifugation, activated T cells were added to each well and incubated at 37℃ with 5% CO_2. Cells were then cultured in and replenished with fresh complete X-VIVO containing cytokines every 2–3 days. After 7 days, beads were magnetically removed, and cells were further expanded in complete X-VIVO containing cytokines to achieve desired cell yield.

T7EN1 cleavage assay and sequencing

Cells were harvested and digested with 100 μg/ml Proteinase K in lysis buffer (10 μM Tris-HCl, 0.4 M NaCl, 2 μM EDTA and 1% SDS). Genomic DNA was extracted by phenol-chloroform and alcohol precipitation. The T7EN1 cleavage assay was performed as follows: briefly, targeted regions of ICAM-1 and IFNGR2 were PCR-amplified from genomic DNA using 2 × Taq Master Mix (Vazyme) and the products were purified with a PCR cleanup kit (Axygen). Purified PCR product was denatured and re-annealed in NEBuffer 2 (NEB) using a ProFlex 3 x 32-Well PCR System (Applied Biosystems). Hybridized PCR products were digested with T7EN1(NEB) for 30 min and separated by 2% agarose gel. Primers for PCR are listed in Supplementary Table1.

Western blot analysis

Cells were harvested and lysed with RIPA lysis buffer containing a protease inhibitor cocktail (Selleck Chemicals). The lysates were centrifuged for 10 min at maximum speed at 4 °C and the supernatants were mixed with sample loading buffer (5×) and boiled for 5 min. For immunoblotting, proteins from whole-cell lysates were resolved by 10 or 12% sodium dodecyl sulfate-polyacrylamide gel electrophoresis (SDS-PAGE) and then transferred to PVDF membranes. The membrane was blocked for 1 h at 25 °C using 5% non-fat milk and incubated in the presence of anti-IFNGR2 overnight at 4 °C with gentle rocking. The membrane was then washed three times with TBS-T buffer and incubated with horseradish peroxidase-conjugated secondary anti-mouse IgG antibody in 5% non-fat milk TBS-T buffer for 1 h at 25 °C. The membrane was washed three times with TBS-T buffer. PVDF membranes were then exposed for an enhanced chemiluminescence assay using the Gel imaging instrument

(BIO-RAD ChemiDoc MP). α-Tublin was used as a loading control. The gray value of all the bands is measured by ImageJ.

Quantitative PCR

Total mRNA from cultured T cells was isolated by RNeasy Mini Kit (Qiagen Inc). cDNA was then synthesized via PrimeScript 1st Strand cDNA Synthesis Kit (Takara Bio Inc). Quantitative PCR was performed using SYBR Green PCR Master mix (Applied Biosystems) in a reverse transcription PCR (RT-PCR) system (Thermo Fisher Scientific). The comparative Ct values of genes of interest were normalized to the Ct value of β-actin. Then, the 2-Δct method was used to determine the relative expression of the genes, while the 2-ΔΔct method was used to calculate the fold changes of gene expression over control.

Flow cytometry

To determine HER2,mesothelin,ICAM-1,and PD-L1 expression on tumor cells, 1×10^5 cells were labeled in 100 μL FACS buffer (Dulbecco's phosphate-buffered saline) containing 2% FBS) containing HER2 (PE, clone 24D2; Biolegend) , mesothelin (clone 420411 R&D Systems) ,ICAM-1(PE, clone HA58; Biolegend) and PD-L1 (PE, clone
29E.2A3; Biolegend) antibodies for 30 minutes at 4°C in the dark. For analysis of CAR expression, the modified T cells were stained with Biotin-SP conjugated anti-F(ab)2 (Jackson ImmunoResearch, West Grove,PA,USA) followed by PE-labeled streptavidin respectively (BD Biosciences). Expression of coinhibitory receptors was detected via the following fluorochrome-conjugated antibodies: APC anti-human PD-1; PE anti-human LAG-3; APC anti-human TIM-3; T cell function was determined using fluorochrome-conjugated antibodies:PE anti-human CD107a;PE anti-human granzyme B; Alexa Fluor®488 anti-human LFA-1. All monoclonal antibodies were purchased from BioLegend. Acquisition and analysis were performed using a NovoCyte Advanteon with The NovoExpress® software (ACEA Biosciences).

In vitro tumor killing and T cell functional assays

For tumor killing assays, CAR T cells and tumor targets were co-cultured at effector:tumor (E:T) ratios of 1:5 or 1:10 in X-VIVO medium in the absence of exogenous cytokines in 48 or 96-well in a final volume of 400ul or 200 μl per well plates for 24–72 h and analyzed by flow cytometry as described above. All samples were set in triplicate. Tumor cells were plated overnight in the absence or presence IFN-γ prior to addition of T cells. Next, 50 μl of supernatant per well was collected to measure LDH release using a cytotoxicity LDH Assay Kit (Promega) according to the manufacturer's instructions. The cell lysis percentage was calculated as follows: Cytotoxicity (%) = (Experimental -Effector Spontaneous-Target Spontaneous)/(Target Maximum-Target Spontaneous) × 100.

To test for degranulation and cytotoxicity, CAR T cells were incubated with tumor cells for 5 hours in the presence of CD107a antibody and Golgistop protein transport inhibitor (BD Biosciences). After the coculture, cells were harvested, fixed, permeabilized, and stained for intracellular staining. Degranulation (CD107a staining) and intracellular marker staining (Granzyme B) were examined by flow cytometry as described above.

For T cell activation and inhibition assays, CAR-T cells and tumor targets were co-cultured in X-VIVO medium in the absence of exogenous cytokines in 48-well plates for the indicated time points and analyzed by flow cytometry for specific markers of T cell activation.

Stress test

To mimic recursive antigen encounters, we started a coculture of adherent target cells and freshly nonadherent effector cells at E:T=1:10. After 24 (round I) and 48(round II) hours, nonadherent effector cells were moved to a new set of adherent target cells at an indicated E:T ratio.

Neutralization assays

HER2-positive SK-OV-3 tumor cells were seeded at wells of a 96-well U-bottom plate at 1×10^4 cell/200 μl and cultured overnight with or without IFN-γ primed. The next day the media was removed and cells were washed once with PBS. HER2 CAR T cell with fresh medium in the presence of IFN-γ neutralizing antibodies or isotype were then added to the tumor cells, the percentage of cytotoxicity of CAR-T cell was calculated as described above. The supernatants were harvested after 24h cultured and performed the downstream experiment.

Elisa cytokine assays

Supernatants from tumor killing assays were collected at indicated times and frozen at -20°C for further use. The cell-free supernatant was diluted and analyzed for IFN-γ and IL-2 production using a Human IFN-γ ELISA Ready-SET-Go Kit (eBioscience) in accordance with the manufacturer's protocol. Plates were read at 450 nm using a Microplate Reader of Multiskan Ascent (Thermo Scientific) and ascent software 2.06. Values represent the mean of triplicate wells.

Xenograft model of ovarian cancer

B-NSG (NOD-Prkdcscid Il2rgtm1/Bcgen) mice were obtained from Beijing Biocytogen Co.,Ltd, 5-8 week-old NSG mice were maintained under pathogen-free conditions in-house of Sichuan University. In vivo experiments were performed in accordance with the Institutional Animal Care and Use Committee of the State Key Laboratory of Biotherapy, Sichuan University. For in vivo tumor studies, SK-OV-3-Luc(WT), SK-OV-3(IFNGR2 KO) (0.2 million/mouse) were prepared in a final volume of 200 μl medium and engrafted in 6–8 weeks old female B-NSG mice

by intraperitoneal (i.p.) injection and allowed to established tumor for 6 days. Tumor engraftment was monitored by bioluminescence imaging and mice were assigned to treatment groups so that each group possessed equal average tumor luminescence, then received i.p. adoptive transfer of 2 million HER2 CAR-T cells per mouse and IFN-γ(20ug×2) every other day prior to CAR-T cell. Tumor growth was monitored at least once a week via IVIS Bioluminescence imaging (PerkinElmer, Inc., Waltham, MA, USA) and flux signals were analyzed with Living Image software (PerkinElmer). For imaging, mice were i.p. injected with 200 μL D-luciferin potassium salt (Perkin Elmer) suspended in PBS at 3 mg/mouse. Mice were also monitored for survival and sacrificed when the total luminescence of luciferin-treated subject exceeded 1×10^{11} photons/s.

Statistics

Data were presented as mean ± SD. For comparisons between two groups, Student's unpaired two tailed t test were employed. $p < 0.05$ was regarded statistically significant.

References

1. Kutner, Zhang, Reiser. Production, concentration and titration of pseudotyped HIV-1-based lentiviral vectors. *Nature protocols*. **4**,495-505,(2009).

Supplementary Fig.S1

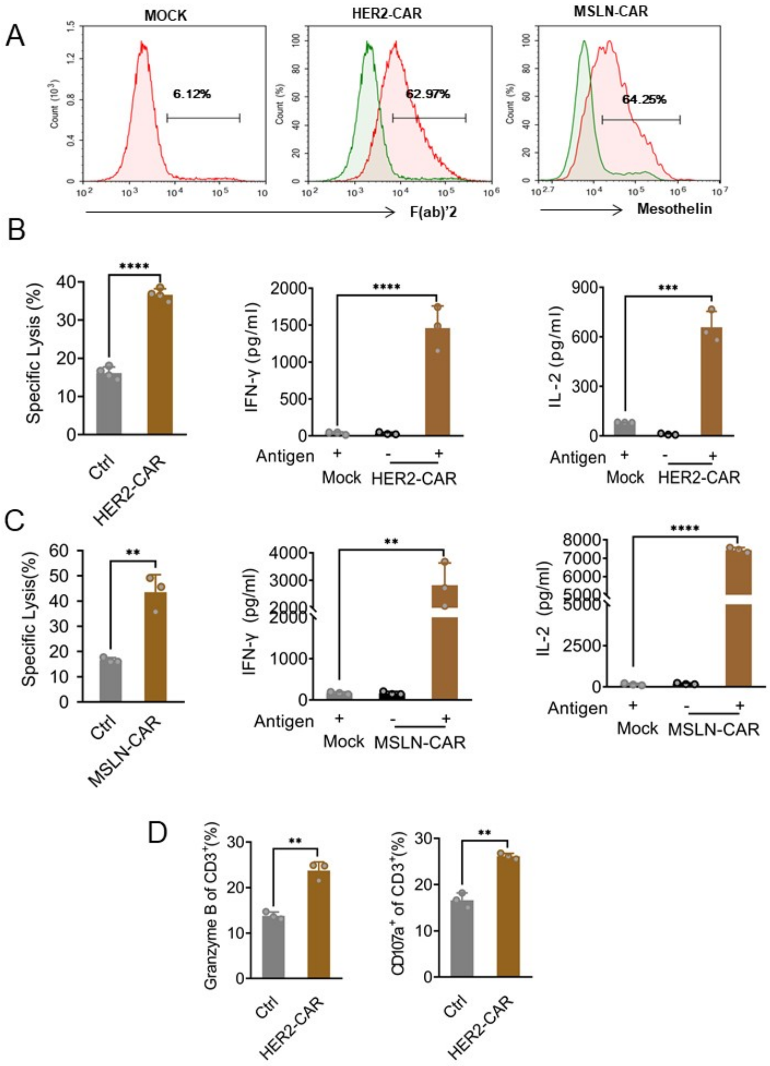

Figure. s1 (**A**) The transduction efficiency of HER2-CAR and MSLN-CAR T cells. (**B-C**) **B** and **C**, the specific cytolytic ability of HER2-CAR and MSLN-CAR T cells to HER2+ target tumor cells SKOV3 were measured by LDH release assays. The secretion of IFN-γ and IL-2 were detected by ELISA assay. (**D**) The frequency of Granzyme B+ or CD107a+ of CD3+ T cells was detected using flow cytometry after HER-CAR T cells were co-cultured with tumor cells for 24 hours. Data are presented as the mean ± SD. $^{*}p < 0.05$, $^{**}p < 0.01$, $^{***}p < 0.001$.

Supplementary Fig.S2

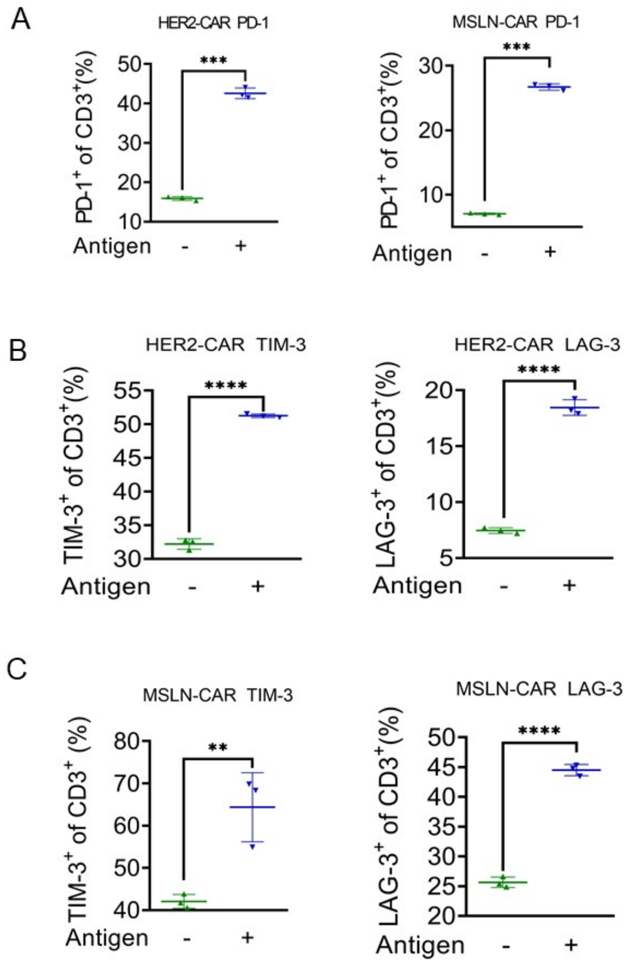

Figure. s2 (**A**) The frequency of PD1^{+} of CD3^{+} T cells was detected using flow cytometry after CAR-T cells were co-cultured with or without tumor cells for 24 hours. **(B-C)** Flow cytometry detection the TIM-3 and LAG-3 expression on HER2 and MSLN CAR T cells respectively after experiencing cognate antigen. Data are presented as the mean ± SD. *p < 0.05, **p < 0.01, ***p < 0.001.

Supplementary Fig.S3

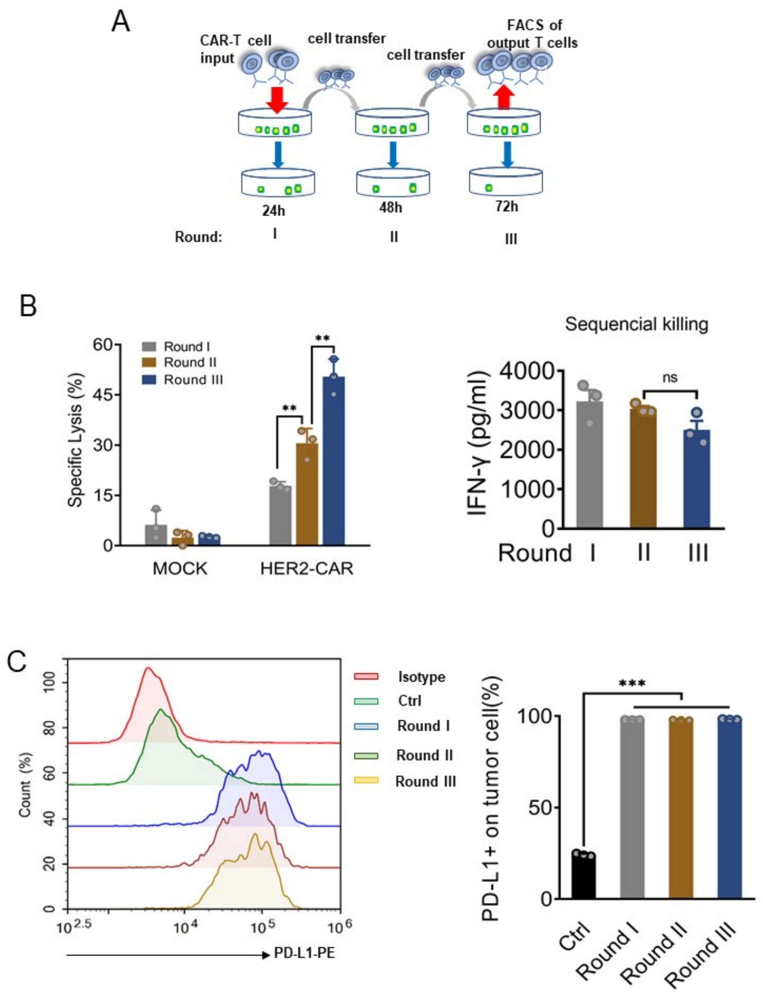

Figure. s3 (**A**) Schema of the serial killing assay. **(B)** The specific lysis of CAR-T to tumor cells in the serial killing were detected by LDH assay. The IFN-γ were quantified by the ELISA. **(C)** Every round of PD-L1 expression on tumor cells was detected by flow cytometry. Data are presented as the mean ± SD. *p < 0.05, **p < 0.01, ***p < 0.001.

Supplementary Fig.S4

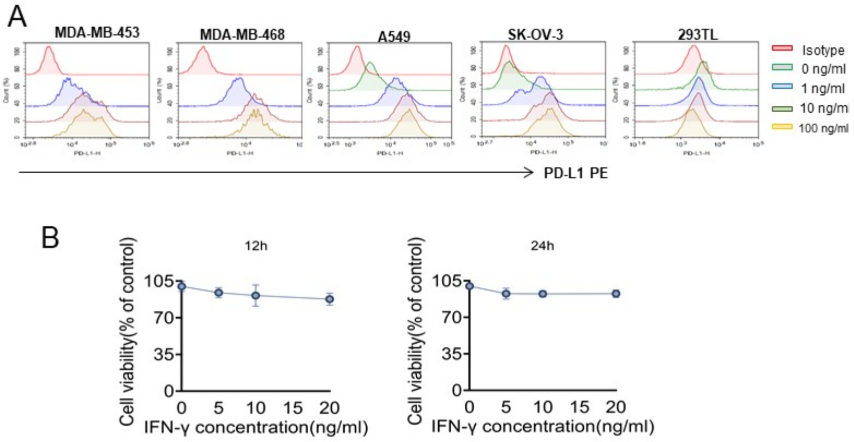

Figure. s4 (**A**) PD-L1 expression on tumor cells stimulated by different concentrations of IFN-γ for 24hrs. **(B)** Quantification of target cell viability following stimulation with different concentrations of IFN-γ ranging from 0-20ng/ml for 12h or 24h. Data are presented as the mean ± SD.

Supplementary Fig.S5

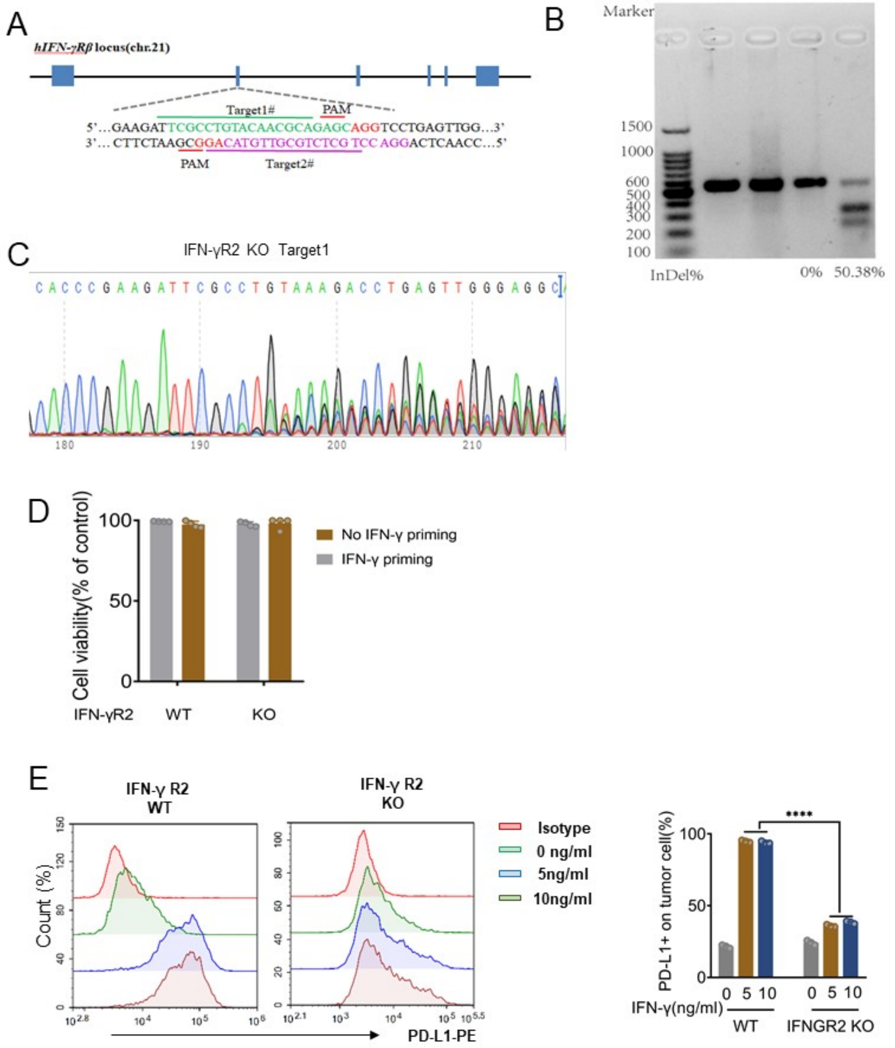

Figure. s5 (A-C) Two sgRNA targeting to IFN-γR2 was designed and the sgRNA location was indicated in the A diagram. the knockout efficiency was determined by T7EN1 cleavage assay (B) and confirmed by sanger sequencing (C). **(D)** Quantification the tumor cell viability with IFN-γR2 wild type or knocked out following IFN-γ priming or not. **(E)** PD-L1 expression induced by different concentrations of IFN-γ on IFN-γR2-KO or control tumor cells was detected by flow cytometry. Data are presented as the mean ± SD. $^*p < 0.05$, $^{**}p < 0.01$, $^{***}p < 0.001$.

Supplementary Fig.S6

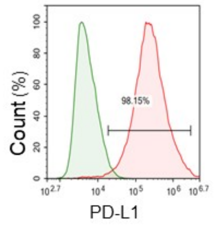

Supplementary Fig.S7

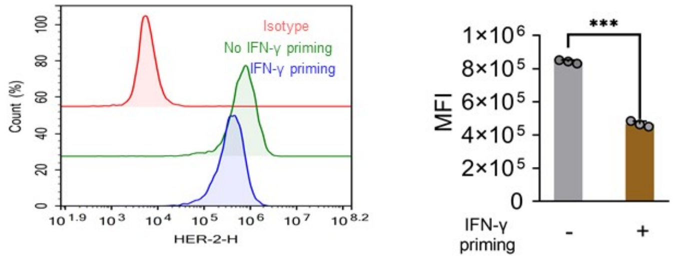

Figure. s6 (A) PD-L1 overexpression on tumor cells was detected by flow cytometry.

Figure. s7 (A) Detection of the HER2 antigen expression on tumor cells by flow cytometry after the IFN-γ stimulation for 24h or not and mean fluorescence intensity of each group was shown in the right. Data are presented as the mean ± SD. ***$p < 0.001$.

Supplementary Fig.S8

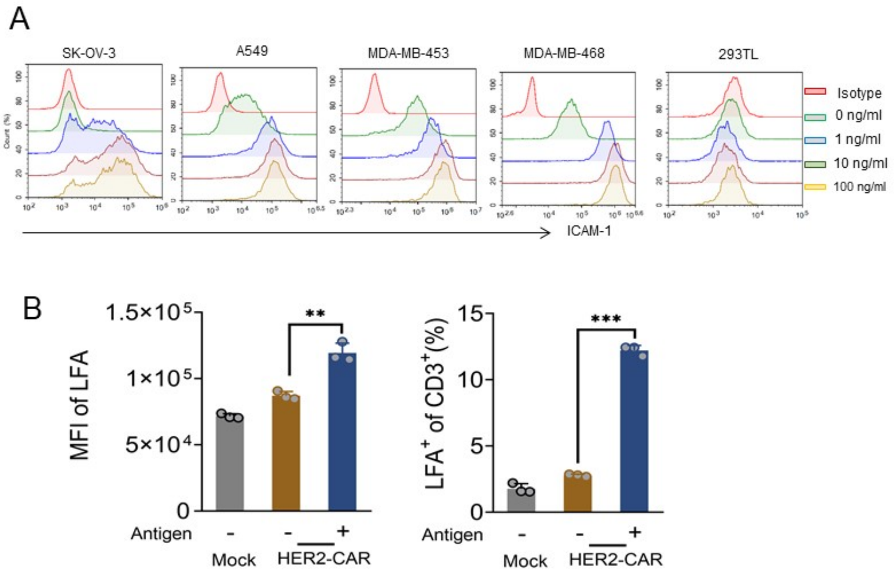

Supplementary Fig.S9

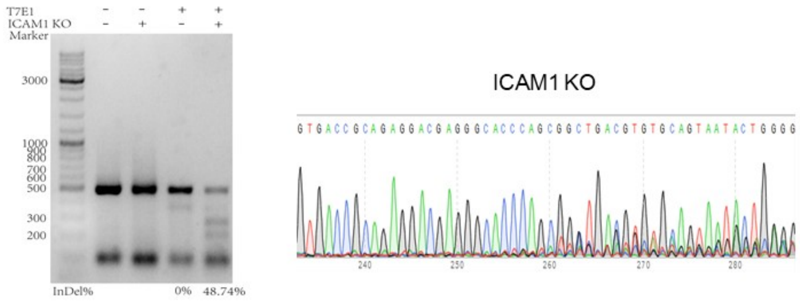

Figure. s8 (A) ICAM-1 expression in tumor cells after IFN-γ stimulation was detected by flow cytometry. **(B)** ICAM-1 and LFA-1 were detected by flow cytometry before and after encountering antigen with different co-culture time.

Figure. s9 (A) ICAM-1 molecule on tumor cells was knocked out using CRISRP-CAS9 technology and confirmed by T7EN1 cleavage assay and sanger sequencing.

Supplementary Fig.S10

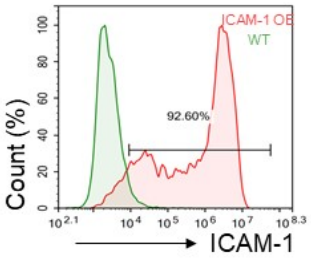

Figure. s10 (A) ICAM-1 overexpressed on tumor cells was confirmed by flow cytometry.

Supplementary Fig.S11

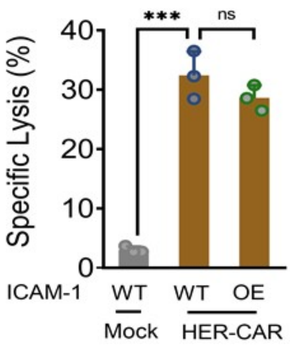

Figure. s11 (A) The specific lysis of CAR-T to tumor cells overexpressing ICAM-1 or not. Data are presented as the mean ± SD. ns: no significance. **p < 0.01, ***p < 0.001.
